# Marginal Bone Level Evaluation after Functional Loading Around Two Different Dental Implant Designs

**DOI:** 10.1155/2016/1472090

**Published:** 2016-11-24

**Authors:** Ko-Ning Ho, Eisner Salamanca, Hsi-Kuai Lin, Sheng-Yang Lee, Wei-Jen Chang

**Affiliations:** ^1^School of Dentistry, College of Oral Medicine, Taipei Medical University, Taipei, Taiwan; ^2^Dental Department of Taipei Medical University, Shuang-Ho Hospital, Taipei, Taiwan; ^3^Dental Department of Taipei Medical University, Wang-Fang Hospital, Taipei, Taiwan

## Abstract

*Purpose*. To investigate peri-implant alveolar bone changes using periapical radiographs before and after prosthetic delivery in submerged and nonsubmerged dental implants.* Methods*. Digital periapical films of 60 ITI Straumann nonsubmerged dental implants and 60 Xive Dentsply submerged dental implants were taken before, immediately after, and 12 and 24 weeks after the prosthetic restoration was delivered.* Results*. The 60-nonsubmerged dental implant group showed mean marginal bone resorption at baseline of 0.10 ± 0.23 mm and 24 weeks later, marginal bone resorption was 0.16 ± 0.25 mm. The submerged dental implant group showed a significantly higher distal marginal bone resorption over the mesial side. Mean marginal bone resorption at baseline was 0.16 ± 0.32 on the mesial and 0.41 ± 0.56 on the distal side. Twenty-four weeks later, it was 0.69 ± 0.69 mm on the mesial and 0.99 ± 0.90 mm on the distal side.* Conclusion*. First, it was possible to determine that submerged implants had a higher mean marginal bone resorption and less bone-to-implant contact than nonsubmerged implants. And second, the distal side of submerged dental implants presented higher marginal bone loss than the mesial side.

## 1. Introduction

In 1965, Brånemark began dental implant placement in edentulous patients, while titanium implants started to be widely exploited in dental treatment [1, 2]. Adell et al. in 1969 introduced for the first time the concept of osseointegration and later redefined it in 1981 as a direct functional and structural connection between living bone and the surface of a load-carrying implant [3]. Additionally, Zarb and Albrektsson in 1991 reproposed the clinical definition of osseointegration as a process where clinically asymptomatic rigid fixation of alloplastic materials is achieved and maintained in bone during functional loading [4]. It became important to study the osseointegration of dental implants with different characteristics. In recent years, many clinical studies have proven that both submerged and nonsubmerged systems of titanium implants could be used to achieve a long-term osseointegration [5].

Replacing missing teeth with an intraosseous implant has proven to be a predictable and successful treatment option. However, the dental implant is always correlated with 1-2 mm alveolar height changes within the first year from occlusal-loaded prostheses placement. Only if the peri-implant bone loss within the first year is less than 2 mm and no more than 0.2 mm annually in subsequent years the dental implant can then comply thereafter with the definition of a successful implant [6].

The soft tissue components around the dental implant consist of junctional epithelium and connective tissues, which form 3-4 mm of biological soft tissue coverage above the supporting bone of the implant. Studies have pointed out that the corresponding peri-implant alveolar bone height is determined by the implant-abutment junction (IAJ) and the relative position of the alveolar bone; this has shown the ability to limit bone loss if the implant shoulder is located above the alveolar crest [7]. There is no significant crestal bone loss when the implant is completely submerged; however, the resorption process starts once the implant is exposed within the oral environment. Therefore, the biological soft tissue coverage and the IAJ with the position of its inevitable microgap have been considered as the critical factors in bone remodeling around the implant [6].

The high rate of clinical success of the submerged implant was verified more than 30 years ago [3, 6, 8–10]. Ericsson et al.'s animal experimental study in 1996 showed that, from all of the bone loss around dental implants, in a one-step surgery, nonsubmerged dental implants lose approximately 90% bone, whereas there is only approximately 60% bone loss in submerged dental implants. In two-stage surgery, there is approximately 40% bone loss in a submerged dental implant, yet the average bone loss of both submerged and nonsubmerged dental implants is similar according to radiographic analysis [11].

Ericsson et al. also published a 5-year follow-up document in 1997 in which submerged implants were placed at the right mandible of 11 patients and nonsubmerged implants at the left mandible. On 5-year follow-up after functional prosthetic restorations were delivered, mesial and distal marginal bone heights of the implants were measured on periapical radiographs. The results showed that there was no significant difference in marginal bone resorption between these two types of dental implants [12].

A wide range of different dental implant designs has been proposed in the past years, and in all of the designs, there have always been changes in the alveolar bone around the area under functional pressure [13–15]. Therefore, in this study, periapical radiographs were used to investigate the peri-implant alveolar bone changes before and until 24 weeks after prosthetic restoration in nonsubmerged and submerged dental implants.

## 2. Materials and Methods

**Figure 1:**
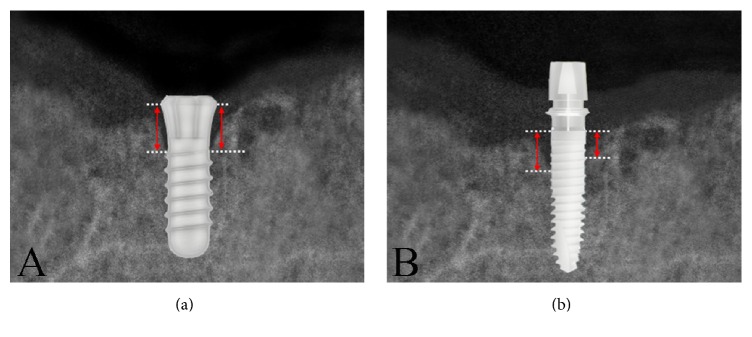
(a) Schematic of ITI Straumann dental implant. Measurement was taken from the cervical line of the implant to the alveolar crest on the mesial and distal aspects. (b) Schematic of an Xive Dentsply dental implant. Measurement was done from the rough-smooth border to the alveolar crest on the mesial and distal aspects.

This is a retrospective case study, where dental implant surgery and prosthetic restoration procedures were performed by the same surgeon and prosthodontist on a total of 97 patients between October 2008 and March 2012 at Shuang-He Hospital (New Taipei, Taiwan). A total of 50 patients received 60 nonsubmerged ITI implants (Straumann AG, Waldenburg, Switzerland) (Figure 1), and the other 47 patients received 60 Xive submerged implants (Dentsply-Friadent, Mannheim, Germany) (Figure 1). Both groups were followed up for more than 24 weeks. During the case collection period, digital periapical radiographies of the dental implants were taken before, immediately after, which was set as the baseline, and 12 and 24 weeks after the prosthetic restoration procedures were delivered.

Among these patients, each received a maximum of two implants. The implants were placed at the position of second premolar, first molar, and second molar. Each implant had to be placed in a different quadrant; the width of the implant was at least 3.8 mm and at least 9.5 mm in length. Patients enrolled were between 25 and 65 years old; these patients had to return for regular follow-up, and periapical radiographs were taken before, immediately after, and 12 and 24 weeks after the prosthetic restoration procedures were delivered. Each patient was to be followed up regularly for at least 24 weeks after the completion of the treatments.

The following exclusion criteria were adopted:

1. Patients with any local or systemic disease.
2. Patients who smoked tobacco.
3. Patients with a betel nut or tobacco chewing habit.
4. Patients with an alcohol drinking habit.
5. Pregnant or breast-feeding patients.
6. Patients on long-term oral medication.
7. Patients who were absent from follow-up.
8. Distance between the implant and the natural tooth which was less than 3 mm or the distance among the implants which was less than 3 mm.
9. Implant-retained overdentures or hybrid dentures which were part of the treatment plan.
10. Bone graft and/or membrane which were used in the surgical procedure.
11. Patients with parafunctional disorders.
12. Patients with poor oral hygiene.

Medical records and previous digital radiographs of the patients were collected. A CCD digital X-ray system (Hyper-X CM) was used; mesial and distal peri-implant bone heights were measured with EZ-Dental professional image recombination software. For the submerged implant system, bone height was measured from the rough-smooth border to the highest point of the proximal bone crest (Figure 1).

Every periapical film was taken with the standard paralleling technique; an X-ray cone indicator was used and patients were instructed to bite on the film. The radiation dose for each periapical film was 60 kV, 6 mA per 0.1 s, radiation exposure time for the premolar area was approximately 0.4 s, and for the molar area it was 0.64 seconds, making patients' radiation exposure for each periapical film 24 mA and 38.4 mA for premolars and molars, respectively.

The EZ-Dental professional image recombination software length calibration tool was utilized to correct the deviation of periapical films, calibration of periapical films was achieved by inputting the actual length of the dental implant, and then, once calibrated the length measuring tool was used to obtain the mesial and distal peri-implant bone height.

After the data were obtained, Student's* t*-test was used to compare the values among the experimental groups, and *P* values <0.05 were considered to indicate statistically significant differences.

## 3. Results

**Figure 2:**
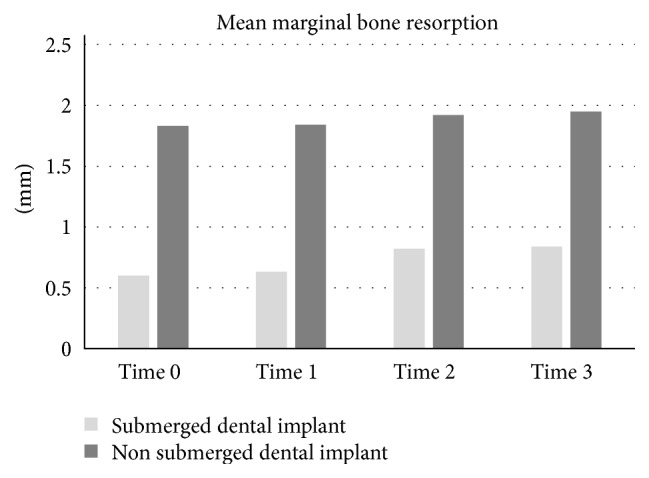
Mean amount of marginal bone resorption of nonsubmerged and submerged dental implant groups. Time 0: before prosthetic procedure delivery, time 1: immediately after delivery, time 2: 12 weeks after delivery, and time 3: 24 weeks after prosthetic procedure delivery.

Out of the total of 60 nonsubmerged implants, there were 20 with a diameter of 4.1 mm and a length of 10 mm; the other 40 had a diameter of 4.8 mm and a length of 10 mm (Table 1). The nonsubmerged implants were implanted in a total of 50 patients, 23 men with a mean age of 51.8 years (range, 37 to 65 years) and 27 women with a mean age of 46 years (range, 30 to 65 years). Mean marginal bone loss can be seen in Figure 2; before prosthetic treatment, delivery was 0.10 ± 0.23 mm, and immediately after prosthetic treatment, it was 0.10 ± 0.23 mm. At 12 weeks after the prosthetic procedure was delivered, it was 0.16 ± 0.25 mm, and at 24 weeks after, it was 0.16 ± 0.25 mm. A comparison of the mean marginal bone resorption at baseline with the mean resorption immediately after prosthetic procedure was delivered showed no difference (0.00 mm), yet there was a difference of 0.09 ± 0.22 mm mean resorption at 12 weeks and 0.12 ± 0.20 mm mean resorption at 24 weeks.

**Figure 3:**
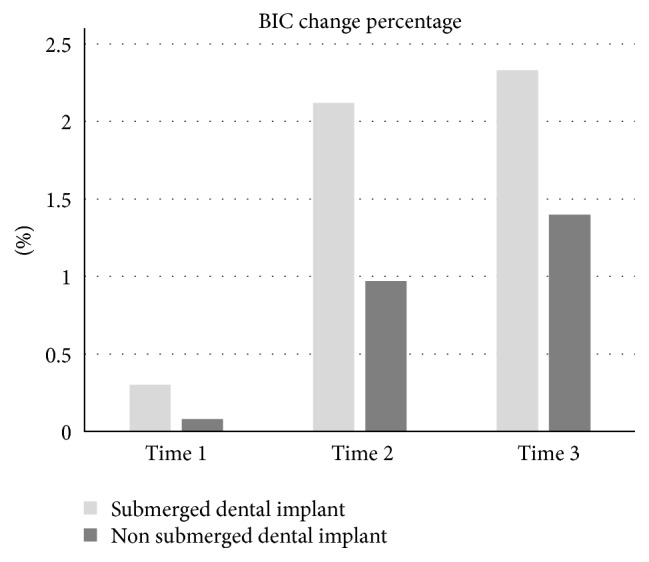
BIC change percentage of nonsubmerged and submerged dental implants. Time 1: immediately after prosthetic delivery, time 2: 12 weeks after delivery, and time 3: 24 weeks after prosthetic procedure delivery.

The bone implant contact (BIC) changes are the percentage of bone resorption at the peri-implant site in contrast to the implant length. The study showed 0.00% immediately after the prosthetic procedure, 0.58%  ±  1.32% 12 weeks after prosthetic delivery, and 0.68%  ±  1.39% at 24 weeks after prosthetic delivery (Figure 3).

In the group of 60 submerged implants, there were 8 implants with a diameter of 3.8 mm and a length of 9.5 mm, 19 implants with a diameter of 3.8 mm and a length of 11 mm, 14 implants with a diameter of 4.5 mm and a length of 9.5 mm, and 19 implants with a diameter of 4.5 mm and a length of 11 mm (Table 1). Of a total of 47 patients, 25 were men with a mean age of 49.8 years (range, 30 to 61 years), and 22 were women with a mean age of 51.5 years (range, 25 to 65 years). Mean marginal bone resorption at baseline was 0.60 ± 0.70 mm, and immediately after prosthetic delivery, it was 0.63 ± 0.71 mm. At 12 weeks after prosthetic delivery, it was 0.82 ± 0.79 mm, and at 24 weeks after prosthetic procedure delivery, it was 0.84 ± 0.81 mm. The difference in the mean peri-implant bone resorption between baseline and immediately after prosthetic procedure delivery was 0.03 ± 0.10 mm, and between baseline and 12 weeks after prosthetic delivery, it was 0.22 ± 0.30 mm. Between baseline and 24 weeks after prosthetic delivery, it was 0.25 ± 0.39 mm. The BIC showed 0.30%  ±  1.28% immediately after prosthetic procedure delivery, 1.09%  ±  1.71% at 12 weeks after prosthetic procedure delivery, and 1.10%  ±  1.74% at 24 weeks after prosthetic procedure (Figure 3).

**Figure 4:**
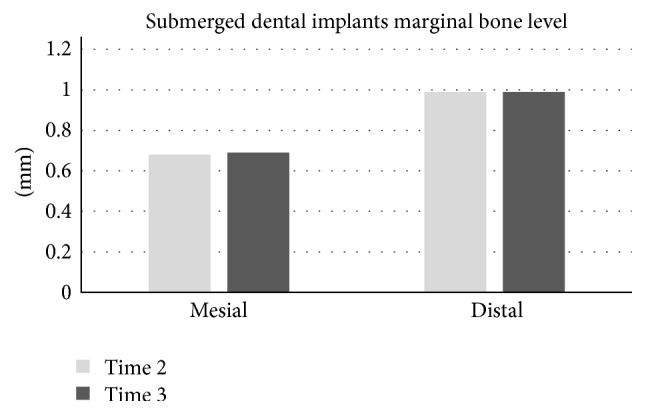
Mean amount of proximal marginal bone resorption of submerged dental implants, a comparison between mesial and distal sides at 12 and 24 weeks after functional loading.

For the submerged implants, mean marginal bone resorption at baseline was 0.16 mm ± 0.32 mm on the mesial side and 0.41 mm ± 0.56 mm on the distal side. Immediately after prosthetic procedure delivery, it was 0.20 mm ± 0.36 mm on the mesial and 0.44 mm ± 0.58 mm on the distal side. At 12 weeks after baseline, it was 0.68 ± 0.68 mm on the mesial and 0.95 mm ± 0.87 mm on the distal side. At 24 weeks after prosthetic procedure delivery, it was 0.69 ± 0.69 mm on the mesial and 0.99 ± 0.90 mm on the distal side. These measurements at four different time points showed a statistically significant difference between mesial and distal marginal bone resorption (Figure 4).

**Figure 5:**
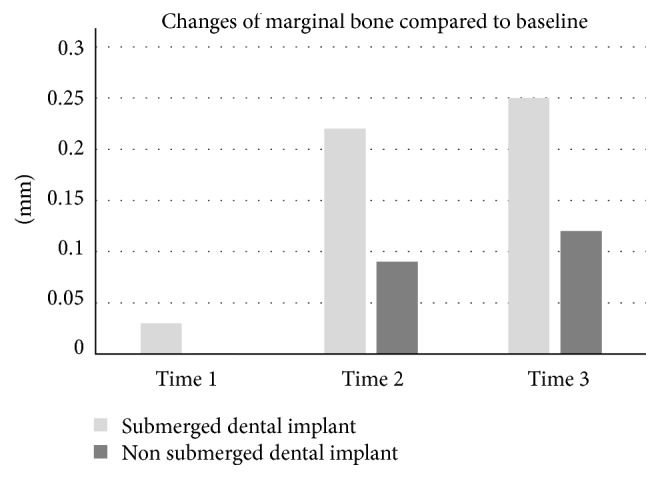
Changes in marginal bone level of nonsubmerged and submerged dental implants. Time 1: immediately after prosthetic delivery (nonsubmerged group at this time did not present any changes), time 2: 12 weeks after delivery, and time 3: 24 weeks after prosthetic procedure delivery.

Figures 3 and 5 represent a comparison between submerged and nonsubmerged implants in the same time frame. As can be seen, submerged implants had a higher mean marginal bone resorption than nonsubmerged implants, and this difference was significantly different regardless of the time point. Additionally, the percentage change in BIC showed a similar pattern.

## 4. Discussion

This study shows that submerged and nonsubmerged implants are both capable of being successfully placed within the oral environment and afford occlusion force with a favorable peri-implant soft tissue reaction. At 12 and 24 weeks after prosthetic delivery, mean marginal bone resorption was less than 0.5 mm in both implant systems. From previous studies, successful osseointegration is identified if there is approximately 1-2 mm of peri-implant bone resorption during the first year [6]. Within the limitations of this study, through radiograph analysis, the two implant systems used in this study were both considered to have successfully osseointegrated and were capable of affording functional occlusion force.

The submerged implant group in this study showed significantly higher marginal bone resorption than the nonsubmerged implant group, regardless of the time point when the periapical film were taken; this may have been due to the need for a second surgery for healing abutment placement, as second flap surgery may lead to additional peri-implant bone resorption. Another possible reason is that the <90° angle between the healing abutment and soft tissue results in susceptible plaque accumulation and limits effective cleaning; these factors may be the causes of higher marginal bone resorption in submerged implants.

The submerged implant group showed no significant difference in marginal bone resorption before and immediately after prosthetic delivery; this can be attributed to the rough surface design of the dental implant, as Nickenig et al. (2009) determined that implants with a rough surface design caused minimal changes in crestal bone levels [16].

According to a study published by Himmlová et al. in 2004, finite element analysis was exploited to compare the effects of varied implant diameter and varied implant length on stress distribution around the cervical area; the results showed 31.5% stress reduction when the implant diameter increased from 3.6 mm to 4.2 mm, whereas there was only 16.4% stress reduction when the implant diameter increased to 5.0 mm. Likewise, there was 7.3% stress reduction when the implant length increased from 8 mm to 12 mm [17]. Thereafter, Baggi et al. and Chou et al. published studies in 2008 and 2010, respectively; both verified that an increase in implant diameter is more effective in resisting peri-implant marginal bone resorption [18, 19]. In the present study, the employed nonsubmerged implants had the same length (10 mm) but a different diameter (4.1 mm versus 4.8 mm); because of this, there was no significant difference in marginal bone resorption. There was also no significant difference between the submerged implants employed, even though in this study, different lengths (9.5 mm or 11 mm) and also different diameters (3.8 mm or 4.5 mm) were used within this group.

For the submerged implant group, there was significantly higher marginal bone resorption at the distal side of the implants, regardless of the time point: at baseline and immediately after and 12 or 24 weeks after prosthetic procedure delivery. Norton (1998) radiographically evaluated 33 single-tooth implants in a 4-year follow-up and reported considerably smaller amounts of crestal bone loss: 0.32 mm mesially and 0.34 mm distally between 6 months and one year. The cumulative mean marginal bone loss mesially and distally was 0.42 mm and 0.40 mm from 1 to 2 years, 0.54 mm and 0.43 from 2 to 3 years, 0.51 mm and 0.24 mm from 3 to 4 years, and 0.62 mm and 0.60 mm for implants past their 4-year recall [20]. The study postulated that the significantly low degree of crestal bone loss resulted from a microthreaded crest module and rough surfaces: grit blasted with TiO_2_ particles as well as an internal conical interface [7]. In the present study, the submerged implant was a hydroxyapatite-coated pure titanium implant with a stepped screw design and an internal hexagon as antirotational element in the connection area. Even though the implants were submerged, there were differences between the implant systems. Norton's study showed similar mesial and distal marginal bone resorption between 6 months and 1 year but reported 0.51 mm on the mesial and 0.24 mm on the distal sides from 3 to 4 years without explaining the difference between the two sides, while in the present study, at 6 months, marginal bone loss of 0.69  ±  0.69 mm on the mesial side and 0.99  ±  0.90 mm on distal side was visible, showing more bone resorption at earlier stages and observing more distal than mesial marginal bone resorption. These differences may be because the occlusal forces and difficulty with cleaning in the distal area might have resulted in more plaque accumulation than in the mesial area, especially around the first and second molars. However, the marginal bone resorption from 12 to 24 weeks after prosthetic delivery in the present study had very minimal changes, which indicates that the marginal bone remained stable.

In a more recent study, Burtscher et al. [21] conducted a 7-year prospective radiographic evaluation of marginal bone level around Brånemark and Xive implant systems. Both implant systems were clinically satisfactory. Nevertheless, the Brånemark group showed a better radiological performance than the Xive group [21]. The marginal bone loss immediately after prosthetic delivery was 0.43 mm in mesial and 0.34 mm in the distal area, reaching 1.74 mm in the mesial and 1.62 mm in the distal area at 7 years. The findings of Burtscher et al. are opposite to those of the present study, having more bone loss in mesial than in distal side, even though they used Xive dental implants with the same abutment internal hex connection, but with a different type of prosthodontic restoration. This leads us to believe that the differing behavior between mesial and distal marginal bone loss in submerged implants is due to occlusal forces.

Despite the fact that this study only used periapical radiographs and was carried out in a small time frame, all of the dental implants were considered successful according to the criteria proposed by Albrektsson in 1985 [22]. The clinical implications of the present study are nevertheless that marginal bone resorption was seen in nonsubmerged and submerged dental implants, and a difference was seen between the mesial and distal side of the submerged dental implants, supporting the findings of others who reported this discrepancy [20, 21]. However, none of them were able to determine the reason for such a discrepancy; instead they were only able to explain the marginal bone loss as a whole. This situation concerns the prosthetic rehabilitation of the dental implant and the following criteria are proposed to perform long-term evaluation through periapical radiographs, the most used tool by clinicians to evaluate osseointegration and bone behavior. Making clinicians more concerned about how marginal bone loss can look in a periapical radiography in a treatment's follow-up and distinguishing bone loss from natural remodeling around the dental implants or the beginning of progressive bone destruction.

## 5. Conclusion

Within the limitations of this study, it was first possible to determine that submerged implants had higher mean marginal bone resorption and less bone-to-implant contact than nonsubmerged implants. Second, the distal side in submerged dental implants presented higher marginal bone loss; this kind of bone change needs to be taken into account for future prosthetic treatments plans and their long-term maintenance. Third, further study with longer periods of time of this difference between mesial and distal side marginal bone loss is necessary.

## Figures and Tables

**Table 1:** Dental implant length and width presented in mm.

Width	Nonsubmerged dental implant group	Submerged dental implant group	
	Length		
	10	9.5	11
3.8	—	8	19
4.1	20	—	—
4.5	—	14	19
4.8	40	—	—

Total	60	22	38

## References

[1] Brånemark P. I. (1971). *Intravascular Anatomy of Blood Cells in Man*.

[2] Brånemark P. I. (1972). Rehabilitation with intra-osseous anchorage of dental prosthesis. *Tandläkartidningen*.

[3] Adell R., Lekholm U., Rockler B., Branemark P. I. (1981). A 15-year study of osseointegrated implants in the treatment of the edentulous jaw. *International Journal of Oral Surgery*.

[4] Zarb G., Albrektsson T. (1991). Osseointegration: a requiem for the periodontal ligament. *The International Journal of Periodontics and Restorative Dentistry*.

[5] Lambrecht J. T., Filippi A., Künzel A. R., Schiel H. J. (2003). Long-term evaluation of submerged and nonsubmerged ITI Solid-Screw Titanium implants: a 10-year life table analysis of 468 implants. *International Journal of Oral and Maxillofacial Implants*.

[6] Fickl S., Zuhr O., Stein J. M., Hürzeler M. B. (2010). Peri-implant bone level around implants with platform-switched abutments. *The International Journal of Oral & Maxillofacial Implants*.

[7] Oh T.-J., Yoon J., Misch C. E., Wang H.-L. (2002). The causes of early implant bone loss: myth or science?. *Journal of Periodontology*.

[8] Albrektsson T., Zarb G., Worthington P., Eriksson A. R. (1986). The long-term efficacy of currently used dental implants: a review and proposed criteria of success. *The International Journal of Oral & Maxillofacial Implants*.

[9] Albrektsson T., Zarb G. A. (1993). Current interpretations of the osseointegrated response: clinical significance. *The International Journal of Prosthodontics*.

[10] Adell R., Eriksson B., Lekholm U., Brånemark P. I., Jemt T. (1990). Long-term follow-up study of osseointegrated implants in the treatment of totally edentulous jaws. *The International Journal of Oral & Maxillofacial Implants*.

[11] Ericsson I., Nilner K., Klinge B., Glantz P.-O. (1996). Radiographical and histological characteristics of ssubmerged and nonsubmerged titanium implants. An experimental study in the Labrador dog. *Clinical Oral Implants Research*.

[12] Ericsson I., Randow K., Nilner K., Petersson A. (1997). Some clinical and radiographical features of submerged and non-submerged titanium implants: a 5-year follow-up study. *Clinical Oral Implants Research*.

[13] Buser D., Mericske-Stern R., Bernard J. P. (1997). Long-term evaluation of non-submerged ITI implants. Part 1: 8-year life table analysis of a prospective multi-center study with 2359 implants. *Clinical Oral Implants Research*.

[14] Collaert B., De Bruyn H. (1998). Comparison of Brånemark fixture integration and short-term survival using one-stage or two-stage surgery in completely and partially edentulous mandibles. *Clinical Oral Implants Research*.

[15] Bozkaya D., Muftu S., Muftu A. (2004). Evaluation of load transfer characteristics of five different implants in compact bone at different load levels by finite elements analysis. *The Journal of Prosthetic Dentistry*.

[16] Nickenig H.-J., Wichmann M., Schlegel K. A., Nkenke E., Eitner S. (2009). Radiographic evaluation of marginal bone levels adjacent to parallel-screw cylinder machined-neck implants and rough-surfaced microthreaded implants using digitized panoramic radiographs. *Clinical Oral Implants Research*.

[17] Himmlová L., Dostálová T., Kácovský A., Konvičková S. (2004). Influence of implant length and diameter on stress distribution: a finite element analysis. *The Journal of Prosthetic Dentistry*.

[18] Baggi L., Cappelloni I., Di Girolamo M., Maceri F., Vairo G. (2008). The influence of implant diameter and length on stress distribution of osseointegrated implants related to crestal bone geometry: a three-dimensional finite element analysis. *The Journal of Prosthetic Dentistry*.

[19] Chou H.-Y., Müftü S., Bozkaya D. (2010). Combined effects of implant insertion depth and alveolar bone quality on periimplant bone strain induced by a wide-diameter, short implant and a narrow-diameter, long implant. *The Journal of Prosthetic Dentistry*.

[20] Norton M. R. (1998). Marginal bone levels at single tooth implants with a conical fixture design. The influence of surface macro- and microstructure. *Clinical Oral Implants Research*.

[21] Burtscher D., Norer B., Dalla Torre D., Beier U., Schubert K., Grunert I. (2015). A 7-year prospective radiographic evaluation of marginal bone level around two different implant systems: a randomized clinical trial. *Clinical Oral Implants Research*.

[22] Albrektsson T., Isidor F., Lang N. P. Consensus report of Session IV.

